# Quinones from *Cordia* species from 1972 to 2023: isolation, structural diversity and pharmacological activities

**DOI:** 10.1007/s13659-023-00414-y

**Published:** 2023-11-24

**Authors:** Rostanie Dongmo Zeukang, Jarmo-Charles Kalinski, Babalwa Tembeni, Eleonora D. Goosen, Jacqueline Tembu, Turibio Tabopda Kuiate, Dominique Serge Ngono Bikobo, Maurice Tagatsing Fotsing, Alex de Théodore Atchadé, Xavier Siwe-Noundou

**Affiliations:** 1 Department of Organic Chemistry, Faculty of Science, University of Yaounde I, PO Box 812, Yaounde, Cameroon; 2 Department of Biochemistry and Microbiology, Faculty of Science, Rhodes University, PO Box 94, Makhanda, 6140 South Africa; 3 Department of Pharmaceutical Sciences, School of Pharmacy, Sefako Makgatho Health Sciences University, Medunsa, PO Box 218, Pretoria, 0204 South Africa; 4 Division of Pharmaceutical Chemistry, Faculty of Pharmacy, Rhodes University, PO Box 94, Makhanda, 6140 South Africa; 5 Department of Chemistry, Tshwane University of Technology, Private Bag X680, Pretoria, 0001 South Africa

**Keywords:** *Cordia*, Boraginaceae, Quinones, Meroterpenoids, Biogenesis, Pharmacological activities

## Abstract

Plants of the genus *Cordia* (Boraginaceae family) are widely distributed in the tropical regions of America, Africa, and Asia. They are extensively used in folk medicine due to their rich medicinal properties. This review presents a comprehensive analysis of the isolation, structure, biogenesis, and biological properties of quinones from *Cordia* species reported from 1972 to 2023. Meroterpenoids were identified as the major quinones in most *Cordia* species and are reported as a chemotaxonomic markers of the *Cordia*. In addition to this property, quinones are reported to display a wider and broader spectrum of activities, are efficient scaffold in biological activity, compared to other classes of compounds reported in *Cordia*, hence our focus on the study of quinones reported from *Cordia* species. About 70 types of quinones have been isolated, while others have been identified by phytochemical screening or gas chromatography. Although the biosynthesis of quinones from *Cordia* species is not yet fully understood, previous reports suggest that they may be derived from geranyl pyrophosphate and an aromatic precursor unit, followed by oxidative cyclization of the allylic methyl group. Studies have demonstrated that quinones from this genus exhibit antifungal, larvicidal, antileishmanial, anti-inflammatory, antibiofilm, antimycobacterial, antioxidant, antimalarial, neuroinhibitory, and hemolytic activities. In addition, they have been shown to exhibit remarkable cytotoxic effects against several cancer cell lines which is likely related to their ability to inhibit electron transport as well as oxidative phosphorylation, and generate reactive oxygen species (ROS). Their biological activities indicate potential utility in the development of new drugs, especially as active components in drug-carrier systems, against a broad spectrum of pathogens and ailments.

**Graphical Abstract:**

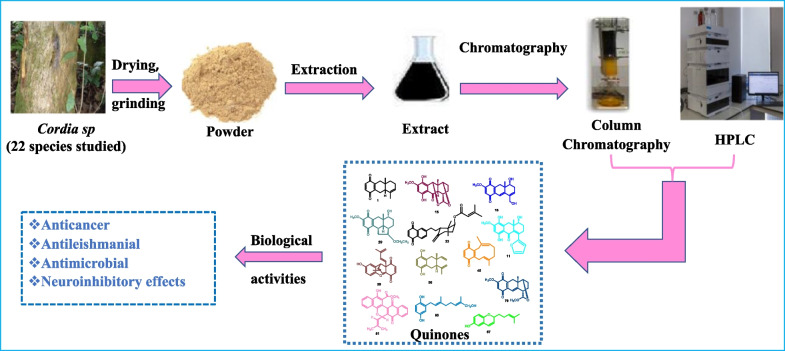

## Introduction

Many plants are traditionally used to treat human diseases, including plants from the genus *Cordia* [1]. *Cordia* is among the largest genera in the Boraginaceae family [2–4], with around 300 identified species [2, 5, 6]. Medicines prepared from these plants are commonly used to treat pains, digestive system and blood disorders, urogenital infections, influenza, cardiac and vascular diseases, coughs, asthma, inflammation, worm infestation, ringworm [7–10], syphilis, as well as dermal and mucosal lesions [11]. The medical utilization of different parts (leaves, stem, stem bark, roots, flowers, and fruits) of *Cordia* species is due to the presence of diverse bioactive constituents, such as terpenoids [9, 12], cinnamates [13], flavonoids [14], pyrrolizidine alkaloids [15]. *Cordia* species are a source of natural products with an extensive range of pharmacological activities, including antimalarial, antioxidant, antiviral, and wound healing properties [9, 16]. They are promising sources for discovering and developing new drug formulations. Apart from their pharmacological application in folk medicine, they are grown as ornamental plants [7], and their wood is used for construction work, boat and furniture building [17–19]. The genus is known for producing a great diversity of quinone natural products, which are often found to be major phytochemical components, especially in extracts from the heartwood and roots [8].

Quinones have long been considered one of the important natural product classes in developing new drugs due to their valuable biological properties such as antioxidant, anti-inflammatory [20], antimalarial, antibacterial, antifungal, and anticancer activities [21, 22]. They have the ability to exist in several redox states, can be highly reactive and play a major role in oxidative mechanisms [23]. Moreover, they are able to elicit oxidative DNA cleavage [24]. Exemplary mitomycin C, a chemotherapy drug used for the treatment of tumors, was isolated from cultures of the bacterium *Streptomyces caespitosus* in 1958 [25]; daunorubicin, an anthraquinone isolated from the soil bacterium *Streptomyces peucetius* in 1963 is known for its potent antileukemic effect; a close analogue, doxorubicin, was isolated from the same strain in 1969 and is used to treat a variety of malignant tumors [26, 27]; vitamin K, a naphthoquinone derivative, is indicated to improve blood coagulation [28]. Furthermore, oncocalyxone A, a benzoquinone isolated from *Cordia oncocalyx* and tested in vivo and in vitro models, showed a large spectrum of pharmacological uses such as antiproliferative/cytotoxic activities against mammalian cells, anti-inflammatory, neuroinhibitory and analgesic effects, as well as antimicrobial and antibiofilm activities [26]. Previous studies have also reported that *Cordia* quinones exhibited pharmacological activities such as antimalarial, antifungal, antimycobacterial and larvicidal activities in addition to cytotoxicity against mammalian cell lines [4, 17, 29–31].

Quinones occurring in *Cordia* species are primarily classified as meroterpenoid benzoquinones, meroterpenoid hydroquinones, and meroterpenoid naphthoquinones [17, 32–34]. Moreover, literature reports on their isolation suggested quinones (meroterpenoids and their derivatives) as one of the chemomarkers of *Cordia* genus [4, 5, 32, 34]. Even though numerous meroterpenoid quinones have been isolated from *Cordia* species since 1970, no experimentally verified biosynthetic scheme has been reported [34]. However, logical deductions have led to the proposal of a potential biosynthetic pathway for some meroterpenoid quinones from *Cordia* species [17, 34–112]

Several studies have investigated the phytochemical and biological studies of *Cordia* species, and most reports focused on chemical constituents, their biological activities, and the chemical synthesis of meroterpenoid quinones. Some of this work has been reviewed in previous works. For instance, Oza et al. reviewed the pharmacological uses, isolation and biology activities of compounds and extracts from the *Cordia* genus until 2016 [8]. Furthermore, Matias et al. reviewed ethnopharmacological and ethnobotanical uses of the genus *Cordia* until March 2014 [7]. Most reports discussing quinones of *Cordia* species focus on South American species used in Brazilian folk medicine.

The relevant information about *Cordia* quinones published between 1972 and 2023, their chemistry, structure, biogenesis and pharmacological activities was obtained through online database search using Scifinder (https://scifinder.cas.org), Science Direct (https://www.sciencedirect.com), PubMed (https://pubmed.ncbi.nlm.nih.gov), and Google Scholar (https://scholar.google.com). The search terms were the following keywords and combinations: *Cordia* species, quinone compounds, meroterpenoids, biosynthesis, biogenesis, and pharmacological activities. The search results thus obtained were critically reviewed for the descriptions of previously described *Cordia* quinones regarding their structure, biogenesis, biological activities, the occurrence of their source organisms, the extraction and purification protocols employed, and the plant parts used. Additional information was obtained by reviewing the cited references in the selected articles.

## Occurrence of *Cordia* quinones

Quinones are a diverse natural product class biosynthesized by plants, fungi, algae, and bacteria [38], and numerous protocols for their chemical synthesis were reported [39]. They are characterized by *ortho*- or *para*-dione substituted cyclic aromatic systems as found in benzoquinones or condensed polycyclic aromatic systems [20] exemplified by naphthoquinones, anthraquinones, and phenanthraquinones [20, 21].

Quinones are biosynthesized in plants via different metabolic pathways with diverse precursors. These include acetate-polymalonate, aromatic amino acids, shikimic acid-*o*-succinoylbenzoic acid, and mevalonic acid pathways [40]. They play an essential part in physiological and enzymatic systems due to their principal role as redox agents in many electron-transfer processes in living organisms [21, 41].

Up to 2023, approximately 70 quinones were isolated from *Cordia* species consisting mainly of meroterpenoid quinones, the principal quinone type isolated from this genus. Additionally, meroterpenoid quinones were identified by GC–MS profiling of different extracts of *Cordia rothii* [42] and by chromatographic fingerprint analysis of bark dichloromethane extract and hexane leaf extract of *Cordia dodecandra* using UV-DAD HPLC [10].

Meroterpenoids are a class of natural products derived partially from terpenoid and quinone biosynthetic pathways [43, 44], where terpenoid and aromatic quinone moieties are linked by carbon–carbon (C–C) and carbon–oxygen (C–O) bonds [45]. Meroterpenoids have been isolated from animals, fungi, marine organisms (algae, microorganisms and invertebrates), and higher plants [46, 47]. Meroterpenoids exhibit a great diversity of structures. These can be a simple molecular structure comprising a prenyl unit linked to a phenolic derivative moiety such as hydroquinone or more complex structures by ring cyclization and chain rearrangement of various length terpenoid side chains [46, 48].

Terpenoids are broadly classified into two major groups depending on their biosynthetic origins:

Firstly, polyketide-terpenoids are grouped according to the number of acyl units that are incorporated to form the polyketide chain (originating from successive condensation of simple carboxylic acids under the control of the polyketide synthases (PKSs)) and the mode of cyclization present. [43, 48]. Polyketide meroterpenoids can have a tri-, tetra- or polyketide chain connected to the terpenoid moiety [48].

Secondly, non-polyketide-terpenoids in which quinones, protocatechuic acid derivatives, dehydroquinic acid or related subunits originating from shikimate pathways are joined to a terpenoid skeleton by a single carbon–carbon (C–C) bond [43].

Previous chemical studies of meroterpenoids revealed that their purification usually follows maceration and conventional extraction methods using organic solvents or their aqueous mixtures [48]. The macerated raw material was extracted with methanol and aqueous methanol (80%) [49–53]; ethanol and aqueous ethanol (70–95%) [54–56]; ethyl acetate [57–61] and petroleum ether [62]. Crude extracts are commonly fractioned by liquid–liquid extraction (hexane; chloroform or dichloromethane, ethyl acetate and butanol) [49, 51, 54, 63]; and purify by silica gel column chromatography (CC) (*n*-Hexane–ethyl acetate; *n*-hexane–acetone; cyclohexane-dichloromethane-methanol gradient; petroleum ether; ethyl acetate; isooctane-ethyl acetate–methanol; ethyl acetate–methanol [49, 56, 61–65]; Sephadex LH-20 CC (Dichloromethane-methanol (1:1); chloroform–methanol (3:2); methanol) [65–69]; MCI gel CHP20P CC (water–methanol (20–100%); methanol–water (60–100%) [55, 67, 68, 70] and RP-HPLC (acetonitrile—0.01% trifluoroacetic acid, 88:12 (v/v); acetonitrile–water (80:20–100:0); methanol–water 25%) [55, 66, 67, 71].

The present summarizes quinones from 25 *Cordia* species, among which meroterpenoid quinones were present in 22 species. The summary of various types of isolated meroterpenoid quinones from these 22 *Cordia* species and their biological activities are listed in Table 1.

**Table 1:** Quinones and their biological studies

Species name	Class (*n* = number of isolated compounds)	Biological study IC_50_/MIC (μM)/MIQ (μg)	Reference standard values IC_50_/MIC (μM)/MIQ (μg)	References
*C. abyssinica*	Meroterpenoid benzoquinone (*n* = *3*)	Antileishmanial *L. major* (2.5) Antimalarial (0.2 ± 0.1) Anticancer KB (6.0 ± 0.5) BC-1 (6.4 ± 0.8) NCI-H187 (0.4 ± 0.009) Vero cell line (1.7 ± 0.6)	Amphotericin B (< 0.1) Dihydroartemisinin (0.0012) Ellipticine 0.2 0.2 0.3 0.4	[17, 72, 73]
*C. alliodora*	Meroterpenoid hydroquinone (*n* = *7*)	Antifungal *C. cucumerinum* 15 antileishmanial *L. major* (4.5)	Nystatin 1 Amphotericin B (< 0.1)	[17, 29, 30, 36, 74]
*C. americana (Patagonula americana)*	Meroterpenoid benzoquinone (*n* = *1*) Meroterpenoid hydroquinone (*n* = *1*)	–	–	[75]
*C. elaeagnoides*	Meroterpenoid hydroquinone (*n* = *5*)	Antimalarial, 3.6 ± 0.1	Dihydroartemisinin (0.0012)	[17, 76]
*C. corymbosa*	Meroterpenoid naphtoquinone (*n* = *4*)	Antifungal *C. albicans* 3 *D. cucumerinum* 3	Nystatin 1 1	[77, 78]
*C. curassavica*	Meroterpenoid naphtoquinone (*n* = *4*)	Antifungal *C. albicans* 3 *D. cucumerinum* 3 Larvicidal *Aedes aegypti* 25	Nystatin 1 1 Plumbagin 6.25	[28]
*C. fragrantissima*	Meroterpenoid benzoquinone (*n* = *4*) Meroterpenoid hydroquinone (*n* = *4*)	Antileishmanial *L. major* (4.1)	Amphotericin B (< 0.1)	[73, 79]
*C. gerascanthus*	Meroterpenoid benzoquinone (*n* = *3*)	Antileishmanial, *L. panamensis* (5.5) Antimalarial 0.2 ± 0.1	Amphotericin B (< 0.1) Dihydroartemisinin (0.0012)	[17, 72, 73]
*C. gharaf*	Meroterpenoid benzoquinone (*n* = *3*)	Antimalarial 0.2 ± 0.1 Antimycobacterial 1.5	Dihydroartemisinin (0.0012) Rifampicin 0.0047	[ 17, 72]
*C. glazioviana*	Meroterpenoid benzoquinone (*n* = *1*) Meroterpenoid hydroquinone (*n* = *4*) Meroterpenoid naphtoquinone (*n* = *1*)	Anti-inflammatory (RAW 264.7) 50.34 ± 9.88	Dexamethasone 1.7 ± 0.04	[34]
*C. globifera*	Meroterpenoid benzoquinone (*n* = *4*) Meroterpenoid hydroquinone (*n* = *2*)	Antimycobacterial 6.2 Antimalarial 2.1 ± 0.5 Anticancer NCI-H187 (0.5 ± 0.04)	Rifampicin 0.0047 Dihydroartemisinin (0.0012) Ellipticine 0.3	[17, 80]
*C. globosa*	Meroterpenoid benzoquinone (*n* = *1*) Meroterpenoid hydroquinone (*n* = *2*)	Anticancer B-16 (1.30) CEM (1.24) HL-60 (1.56)	Doxorubicin 0.03 0.02 0.02	[5, 31]
*C. goeldiana*	Meroterpenoid benzoquinone (*n* = *4*) Meroterpenoid hydroquinone (*n* = *1*) Meroterpenoid naphtoquinone (*n* = *1)*	Antileishmanial *L. panamensis* (5.5) Anticancer KB (1.5 ± 0.1) BC-1 (1.8 ± 0.1) NCI-H187 (0.2 ± 0.006) Vero cell line (1.4 ± 0.4)	Amphotericin B (< 0.1) Ellipticine 0.2 0.2 0.3 0.4	[72, 73, 79]
*C. leucocephala*	Meroterpenoid naphtoquinone (*n* = *2*)	–	–	[3]
*C. linnaei*	Meroterpenoid naphtoquinone (*n* = 6)	Antifungal *C. albicans* 6 *D. cucumerinum* 3 Larvicidal *Aedes aegyphti* 25	Nystatin 1 1 Plumbagin 6.25	[81]
*C. millenii*	Meroterpenoid benzoquinone (*n* = *4*) Meroterpenoid hydroquinone (*n* = *1*) Meroterpenoid naphtoquinone (*n* = *1*)	Antimalarial 0.2 ± 0.1 Antimycobacterial 1.5 Anticancer KB (1.5 ± 0.1) BC-1 (1.8 ± 0.1) NCI-H187 (0.2 ± 0.006) Vero cell line (1.4 ± 0.4) Antileishmanial *L. panamensis* (5.5)	Dihydroartemisinin (0.0012) Rifampicin 0.0047 Ellipticine 0.2 0.2 0.3 0.4 Amphotericin B (< 0.1)	[17, 33, 73, 79]
*C. monoica*	Meroterpenoid benzoquinone (*n* = *2*)	Antileishmanial *L. major* (2.5)	Amphotericin B (< 0.1)	[72, 73]
*C. oncocalyx (Auxemma oncocalyx)*	Meroterpenoid benzoquinone (*n* = *6*) Meroterpenoid hydroquinone (*n* = *7*) Meroterpenoid naphtoquinone (*n* = *2*)	Neuroinhibitory Cytotoxic PBMC (6.8 ± 3.0) HL-60 (11.2 ± 3.0) CEM (0.76 ± 0.05) Antimicrobial *S. epidermidis* (ATCC 12228™) 9.43	Doxorubicin 1.7 ± 1.1 0.03 ± 0.02 Etoposide (< 1) Vancomycin 1	[32, 82, 83]
*C. platythyrsa*	Meroterpenoid benzoquinone (*n* = *4*) Meroterpenoid hydroquinone (*n* = *1*) Meroterpenoid naphtoquinone (*n* = *1*)	Antileishmanial *L. major* (4.1) Anticancer KB (6.0 ± 0.5) BC-1 (6.4 ± 0.8) NCI-H187 (0.4 ± 0.009) Vero cell line (1.7 ± 0.6)	Amphotericin B (< 0.1) Ellipticine 0.2 0.2 0.3 0.4	[72, 73, 79]
*C. polycephala*	Meroterpenoid naphtoquinone (*n* = *5*)	Anticancer HCT-8 (1.2 ± 1.5) HL-60 (2.2 ± 4.3)	Doxorubicin (0.02 ± 0.03) 0.03 ± 0.05	[4]
*C. rothii*	Meroterpenoid benzoquinone (*n* = *1*) Meroterpenoid hydroquinone (*n* = *3*)	Antimicrobial		[42]
*C. trichotoma*	Meroterpenoid benzoquinone (*n* = *2*)	Antimycobacterial 1.5	Rifampicin 0.0047	[84, 85]

Quinone constituents of *Cordia* species are highly diverse, and continuous phytochemical studies of the roots, stem barks, heartwood, wood, leaves, and whole plant extracts of *Cordia* species led to the isolation and structural identification of various quinone skeletons. The current review reports over 70 quinones (**1**–**70**) obtained from twenty-two *Cordia* species, most of which were isolated from ethanol and *n*-hexane extracts of the roots. These compounds showed significant pharmacological activities, and their biosynthesis has been hypothesized. Their structural elucidation was achieved by mass spectroscopic (MS), 1D and 2D nuclear magnetic resonance (NMR) analysis, chemical derivatization reactions, and X-ray crystallographic analysis. The structures of isolated quinones and their biological activities are summarized in Table 2.

**Table 2:** Reported quinones from *Cordia* species

No	Compound structure and name	Species name	Plant part, extraction solvent	Pharmacological effect, IC_50_ /MIC (μM)/MIQ (μg)/Percentage of inhibition (%)	Positive control IC_50_/MIC (μM)/MIQ (μg)	Reference
**1**		*C. millenii* *C. fragrantissima* *C. abyssinica* *C. gerascanthus* *C. gharaf* *C. goeldiana* *C. monoica* *C. platythyrsa*	Heartwood, CHCl_3;_ Wood, *n*-hexane	Antileishmanial *L. major* (4.1)	Amphotericin B (< 0.1)	[33, 72, 73, 79]
**2**		*C. millenii* *C. globifera* *C. fragrantissima* *C. abyssinica* *C. gerascanthus* *C. gharaf* *C. goeldiana* *C. monoica* *C. platythyrsa*	Heartwood, CHCl_3;_ Roots, *n*-hexane; Wood, *n*-hexane	Antileishmanial *L. major* (2.5) Anticancer KB (6.0 ± 0.5) BC-1 (6.4 ± 0.8) NCI-H187 (0.4 ± 0.009) Vero cell line (1.7 ± 0.6)	Amphotericin B (< 0.1) Ellipticine 0.2 0.2 0.3 0.4	[33, 72, 73, 79]
**3**		*C. millenii* *C. globosa* *C. trichotoma* *C. fragrantissima* *C. abyssinica* *C. gerascanthus* *C. gharaf* *C. goeldiana* *C. platythyrsa* *C. rothii*	Heartwood, CHCl_3;_ Roots, *n*-hexane; Heartwood, EtOH; Wood, *n*-hexane	Antimalarial 0.2 ± 0.1 Antimycobacterial 1.5 Anticancer KB (1.5 ± 0.1) BC-1 (1.8 ± 0.1) NCI-H187 (0.2 ± 0.006) Vero cell line (1.4 ± 0.4) Antileishmanial *L. panamensis* (5.5)	Dihydroartemisinin (0.0012) Rifampicin 0.0047 Ellipticine 0.2 0.2 0.3 0.4 Amphotericin B (< 0.1)	[17, 33, 42, 72, 73, 79, 84]
**4**		*C. millenii* *C. goeldiana* *C. platythyrsa*	Heartwood, CHCl_3_	–	–	[33, 72]
**5**		*C. millenii* *C. goeldiana* *C. platythyrsa*	Heartwood, CHCl_3_	–	–	[33, 72]
**6**		*C. millenii* *C. goeldiana* *C. platythyrsa*	Heartwood, CHCl_3_	–		[33, 72]
**7**		*C. millenii*	Heartwood, CHCl_3_	Antitumor OVCAR-3 (0.8) HepG 2 (1) Antimicrobial (*S.aureus*) 6.25 Anti-inflammatory (Paw edema) 69	Doxorubicin (< 1) Oxacillin 0.39 Indomethacin 67	[33, 72, 86–88]
**8**		*C. oncocalyx*	Sapwood, EtOH	–	–	[32]
**9**		*C. oncocalyx*	Sapwood, EtOH	Neuroinhibitory	–	[32]
**10**		*C. oncocalyx*	Heartwood, EtOH	Neuroinhibitory	–	[32, 82]
**11**		*C. oncocalyx*	Heartwood, EtOH	Neuroinhibitory	–	[32]
**12**		*C. oncocalyx*	Heartwood, EtOH	–	–	[32]
**13**		*C. oncocalyx*	Heartwood, EtOH	–	–	[32]
**14**		*C. oncocalyx*	Heartwood, EtOH	Neuroinhibitory	–	[32, 82]
**15**		*C. oncocalyx* *C. glazioviana*	Heartwood, EtOH	–	–	[32, 34, 82]
**16**		*C. oncocalyx*	Heartwood, EtOH	Neuroinhibitory	–	[32, 82]
**17**		*C. oncocalyx*	Heartwood, EtOH	Neuroinhibitory	–	[32, 82]
**18**		*C. oncocalyx*	Heartwood, EtOH	Neuroinhibitory Cytotoxic PBMC (6.8 ± 3.0) HL-60 (11.2 ± 3.0) CEM (0.76 ± 0.05) Antimicrobial *S. epidermidis* (ATCC 12228™) 9.43 Analgesic	Doxorubicin 1.7 ± 1.1 0.03 ± 0.02 Etoposide (< 1) Vancomycin 1	[32, 83, 85, 92, 93]
**19**		*C. oncocalyx*	Heartwood, EtOH	–	–	[32]
**20**		*C. corymbosa* *C. curassavica*	Roots, *n*-hexane; Roots, CH_2_Cl_2_	Antifungal *C. albicans* 3 *C. cucumerinum* 3 Larvicidal *Aedes aegyphti* 12.5	Nystatin 1 1 Plumbagin 6.25	[30, 77]
**21**		*C. corymbosa* *C. linnaei* *C. polycephala* *C. curassavica*	Roots, *n*-hexane; Roots, CH_2_Cl_2_	Anticancer HL-60 (2.2 ± 4.3) Antifungal *C. albicans* 3 *D. cucumerinum* 3 Larvicidal *Aedes aegyphti* 25	Doxorubicin 0.03 ± 0.05 Nystatin 1 1 Plumbagin 6.25	[4, 30, 77, 81]
**22**		*C. linnaei* *C. corymbosa*	Roots, CH_2_Cl_2;_ Roots, *n*-hexane	–	–	[78, 81]
**23**		*C. corymbosa*	Roots, *n*-hexane	–	–	[78]
**24**		*C. polycephala* *C. linnaei*	Roots, *n*-hexane; Roots, CH_2_Cl_2_	Anticancer HL-60 (8.80 ± 9.30) Antifungal *C. albicans* 6 *D. cucumerinum* 3 Larvicidal *Aedes aegyphti* 12.50 Antileishmanial *L. amazonensis* (4.50 ± 0.30)	Doxorubicin (0.03 ± 0.05) Nystatin 1 1 Plumbagin 6.25 Amphotericin B (0.35 ± 0.05)	[4, 81, 89]
**25**		*C. linnaei*	Roots, CH_2_Cl_2_	Antifungal *C. albicans* 6 *D. cucumerinum* 1.5 Larvicidal *Aedes aegyphti* 50	Nystatin 1 1 Plumbagin 6.25	[81]
**26**		*C. linnaei*	Roots, CH_2_Cl_2_	Antifungal *C. albicans* 6 *D. cucumerinum* 3 Larvicidal *Aedes aegyphti* 25	Nystatin 1 1 Plumbagin 6.25	[81]
**27**		*C. linnaei*	Roots, CH_2_Cl_2_	–	–	[81]
**28**		*C. curassavica* *C. leucocephala*	Roots, CH_2_Cl_2_	Antifungal *C. albicans* 3 *C. cucumerinum* 3 Larvicidal *Aedes aegyphti* 25 Cytotoxic HL-60 (2.7) PBMC (10.4)	Nystatin 1 1 Plumbagin 6.25 Doxorubicin 0.03 1.7	[30, 90]
**29**		*C. curassavica*	Roots, CH_2_Cl_2_	Antifungal *C. albicans* 3 *C. cucumerinum* 3 Larvicidal *Aedes aegyphti* 12.5	Nystatin 1 1 Plumbagin 6.25	[30]
**30**		*C. leucocephala*	Roots, *n*-hexane	Anticancer SF 295 (4.6 ± 5.2)	Doxorubicin 0.4 ± 0.6	[3, 4]
**31**		*C. leucocephala*	Roots, *n*-hexane	–	–	[3]
**32**		*C. polycephala*	Roots, *n*-hexane	Anticancer HL-60 (1.5 ± 2.0)	Doxorubicin (0.03 ± 0.05)	[14]
**33**		*C. polycephala*	Roots, *n*-hexane	Anticancer HCT-8 (1.2 ± 1.5)	Doxorubicin (0.02 ± 0.03)	[4]
**34**		*C. polycephala*	Roots, CHCl_3_	–	–	[3]
**35**		*C. anisophylla*	Roots, CH_2_Cl_2_	Antifungal *C. albicans* (DSY262) ≤ 5 μg	Miconazole 0.0006	[91]
**36**		*C. globifera* *C. alliodora* *C. rothii*	Roots, MeOH; Heartwood, acetone	Antimalarial 0.3 ± 0.0 Anticancer KB (6.9 ± 0.1) BC-1 (3.2 ± 0.2) NCI-H187 1.9 ± 0.1 Vero cell line (1.6 ± 0.4) Antileishmanial *L. major* (4.5)	Dihydroartemisinin (0.0012) Ellipticine 0.2 0.2 0.3 0.4 Amphotericin B (< 0.1)	[17, 36, 42, 73]
**37**		*C. glazioviana*	Heartwood, EtOH	–	–	[34]
**38**		*C. glazioviana*	Heartwood, EtOH	–	–	[34]
**39**		*C. fragrantissima*	Wood, MeOH	Antileishmanial *L. major* (81.4)	Amphotericin B (< 0.1)	[73, 79]
**40**		*C. fragrantissima*	Wood, MeOH	Antileishmanial *L. major* (2.7)	Amphotericin B (< 0.1)	[73, 79]
**41**		*C. fragrantissima*	Wood, MeOH	Antileishmanial *L. major* (> 25)	Amphotericin B (< 0.1)	[79]
**42**		*C. glazioviana*	Heartwood, EtOH	Anti-inflammatory (RAW 264.7) 50.34 ± 9.88	Dexamethasone 1.7 ± 0.04	[34]
**43**		*C. glazioviana*	Heartwood, EtOH	Anti-inflammatory (RAW 264.7) 105.83 ± 5.09	Dexamethasone 1.7 ± 0.04	[34]
**44**		*C. glazioviana*	Heartwood, EtOH	Anti-inflammatory (RAW 264.7) 66.73 ± 10.28	Dexamethasone 1.7 ± 0.04	[34]
**45**		*C. globifera*	Roots, *n*-hexane	Antimycobacterial 6.2 Antimalarial 2.1 ± 0.5 Anticancer NCI-H187 (0.5 ± 0.04)	Rifampicin 0.0047 Dihydroartemisinin (0.0012) Ellipticine 0.3	[17]
**46**		*C. globifera* *C. alliodora* *C. fragrantissima*	Roots, MeOH; Heartwood, acetone; Heartwood, ether; Wood, MeOH	Anticancer KB (12.0 ± 0.2) BC-1 (10.3 ± 0.2) NCI-H187 2.2 ± 0.8 Vero cell line 14.1 ± 1.4 Antileishmanial *L. major* (7.0)	Ellipticine 0.2 0.2 0.3 0.4 Amphotericin B (< 0.1)	[17, 36, 73, 74]
**47**		*C. globifera*	Roots, MeOH	–	–	[80]
**48**		*C. globosa*	Roots, EtOH	–	–	[5]
**49**		*C. globosa*	Roots, EtOH	–	–	[5]
**50**		*C. globosa*	Roots, CH_2_Cl_2_	Anticancer B16 (1.30) CEM (1.24) HL-60 (1.56)	Doxorubicin 0.03 0.02 0.02	[31]
**51**		*C. globosa*	Roots, CHCl_3_	–	–	[31]
**52**		*C. alliodora*	Roots, CH_2_Cl_2_	–	–	[29]
**53**		*C. alliodora*	Heartwood, acetone	–	–	[36]
**54**		*C. alliodora*	Heartwood, acetone	–	–	[36]
**55**		*C. alliodora* *C. rothii*	Heartwood, acetone; Roots, ethyl acetate	–	–	[36, 42]
**56**		*C. alliodora*	Heartwood, acetone	–	–	[36]
**57**		*C. alliodora* *C. rothii*	Heartwood, acetone; Roots, MeOH	–	–	[36, 42]
**58**		*C. oncocalyx*	Wood, EtOH	–	–	[92]
**59**		*C. oncocalyx*	Wood, EtOH	Antiproliferative CEM (1.5 ± 0.3)	Etoposide (< 1)	[93]
**60**		*C. oncocalyx*	Heartwood, EtOH	–	–	[82]
**61**		*C. americana*	Heartwood, chloroform	–	–	[75]
**62**		*C. americana*	Heartwood, chloroform	–	–	[75]
**63**		*C. elaeagnoides*	Heartwood, ether	–	–	[76]
**64**		*C. elaeagnoides*	Heartwood, ether	–	–	[76]
**65**		*C. elaeagnoides*	Heartwood, ether	–	–	[76]
**66**		*C. elaeagnoides* *C. globifera*	Heartwood, ether; Roots, *n*-hexane	Antimalarial 3.6 ± 0.1	Dihydroartemisinin (0.0012)	[17, 76, 79]
**67**		*C. elaeagnoides*	Heartwood, ether	–	–	[76]
**68**		*C. oncocalyx*	Heartwood, EtOH	–	–	[94]
**69**		*C. oncocalyx*	Roots, MeOH	–	–	[94]
**70**		*C. oncocalyx*	Roots, MeOH	– –	–	[94]

Previous studies reported that the wood of *C. dodecandra* used in joinery can cause dermal allergic reactions after prolonged contact [95], and it was explained that the allergy towards woods of *Cordia* species might be due to the presence of cordiachromes [18, 95]. Thus, cordiachromes A (**1**), B (**2**), E (**5**) and F (**6**) from *C. dodecandra* mixed with 1% of petrolatum elicited high sensitization in experimental animals after 48 h and 98 h of exposure [95]. However, another study revealed that cordiachrome F (**6**) had no noticeable effects on human patients after exposure to these mixtures over the same period. Thus, it was suggested that other cordiachromes that were not tested could be the responsible agents causing allergic reactions [18].

## Biogenesis and synthesis of quinones from *Cordia* species

The biosynthesis of meroterpenoid quinones from *Cordia* species has not been experimentally validated, but their biosynthetic sequences have been proposed based on logical deductions. For instance, Moir et al. [33] proposed that cordiachromes (A–F) can be derived from geranyl pyrophosphate and an aromatic precursor unit followed by oxidation of an allylic methyl group and cyclization to *trans,trans*-cylodecatriene. Subsequent acid-catalyzed cyclization led to cordiachromes A (**1**) and B (**2**). *Cis*,*cis*-cylodecatriene afforded cordiachrome C (**3**) via a Cope rearrangement [33]. Cordiachromes D (**4**), E (**5**), and F (**6**) were obtained by methoxylation of the previous cordiachromes, respectively [33]. According to Thomson [45], geranylquinol can be another precursor for cordiachromes. He suggested that geranylquinol may be obtained by oxidative cyclization at a terminal allylic methyl group via allylic alcohol pyrophosphate to provide a cyclodecatriene [45]. Another cyclization of the latter through boat conformation could then conduct to cordiachromes A (**1**) and B (**2**), whereas a cope rearrangement of a cyclodecatriene would lead to cordiachrome C (**3**) [45]. He also suggested that cordiachrome G (**61**) is more optically active than other cordiachromes because the stereospecific allylic oxygenation occurs before the rearrangement of cyclodecatriene [45].

**Scheme 1:**
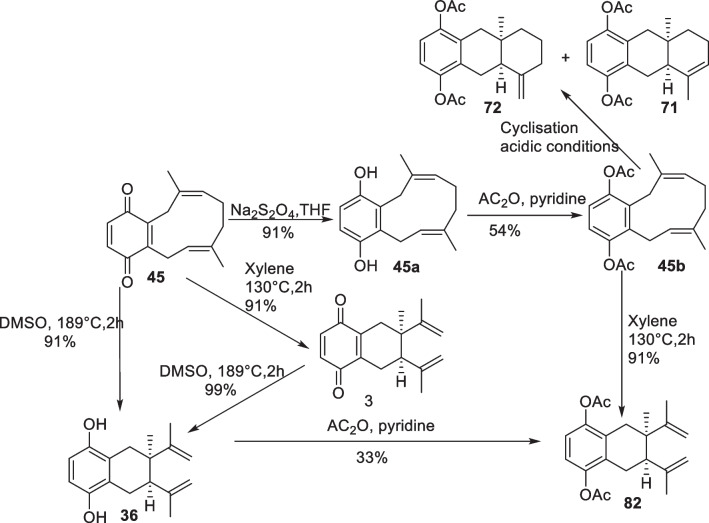
A proposed synthetic pathway for the cordiachrome skeleton [17]

Dettrakul et al. provide information about the biogenesis of cordiachromes. It was suggested that globiferin (**45**), isolated from *Cordia* species, is an intermediate for the biosynthesis of cordiachromes because its structure is similar to *trans,trans*-cylodecatriene proposed by Moir et al. [17]. In addition, the link between the benzoquinone skeleton and the aliphatic chain of globiferin was confirmed by its reduction with Na_2_S_2_O_4_ to dihydroxyglobiferin (**45a**). Cordiachrome C (**3**) was obtained through Cope rearrangement by refluxing compound **45** in xylene. Cordiaquinol C (**36**) was obtained by refluxing compound **45** in DMSO-d*6* for two hours. It was also obtained from cordiachrome C (**3**) under the same conditions. The respective cordiachromes A (**1**) and B (**2**) derivatives, diacetylcordiachromes A (**71**) and B (**72**), were obtained by cyclization of diacetylglobiferin (**45b**) under acidic conditions, were obtained respectively [17]. The suggestions about biosynthesis and synthesis proposed by Dettrakul et al. are resumed in Scheme 1.

According to Matos et al. and Silva et al., meroterpenoid quinones from *Cordia* species are formed via *C*-alkylation of the *p*-hydroxybenzoic acid with prenyl unities which result in the formation of geranyl hydroquinone followed by different chemical reactions such as intramolecular cyclization, oxidation, hydroxylation, *o*-methylation, epoxidation, and decarboxylation [32, 34]. Based on this idea, the biogenesis of the cordiachrome derivatives (**8** to **19**) isolated from *C. oncocalyx* was established [32]. Similarly, the hydroquinones (**37, 38, 42,** and **43**) and naphthoquinones (**15** and **14**) isolated from *C. glazoviana* could follow the same pathway.

It has been suggested that alkannin (**7**), a quinone isolated from *Cordia millenii,* could be biosynthesized from *p*-hydroxybenzoic acid and mevalonate [33]. Leistner, this biosynthetic pathway to form alkannin (**7**) may occur in the Boraginaceae family [40] and, thus, in the *Cordia* genus.

**Scheme 2:**
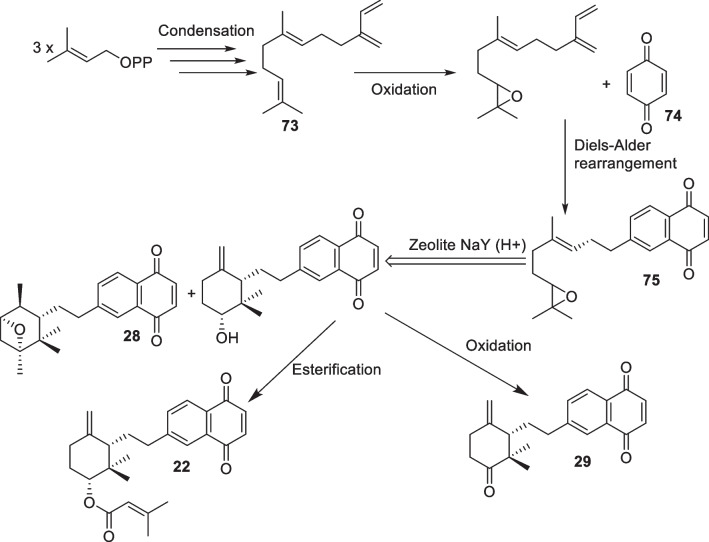
Proposed biosynthesis and synthesis pathway to obtain cordiaquinone skeletons [35, 96]

As for Cordiaquinones biosynthesis, Arkoudis and Stratakis proposed that cordiaquinones are derived from (E)-Naphtoquinone epoxide, their precursor (**75**) which is obtained from E-trans,trans-Farnesol (**73**) and benzoquinone (**74**) through oxidation and Diels–Alder rearrangement, and different cordiaquinones are occurring from precursor through chemical reactions (cyclization, oxidation and esterification) (Scheme 2) [96].

**Scheme 3:**
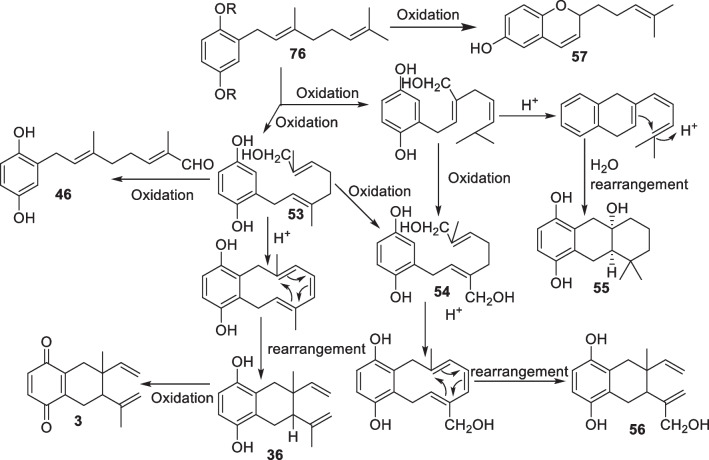
Proposed biosynthesis scheme of *C. alliodora* compounds [36, 37]

Manners and Jurd suggested the biosynthesis of compounds from *C. alliodora*. According to them, the isolation of cordiachromene A (**57**) from *C. alliodora* confirms the presence of geranylphenol (**76**) as a precursor of compounds isolated from *C. alliodora* [36]. They proposed cyclization of the intermolecular geranyl side chain is due to the acid-catalyzed reaction of phenolic nucleus with geranyl C-3 or C-7 allylic hydroxyl group, which afforded to cordallinol (**54**) and alliodorol (**53**), followed by another acid-catalyzed cyclization and intramolecular rearrangement to form cordiol (**55**), cordiaquinols (**36**–**41**), and allioquinol (**56**), which can also be oxidized to cordiachromes (**1**–**6**) and their derivatives (Scheme 3) [36, 37].

According to Manners, *Cordia* compounds could be provided from a geranylphenol precursor that would then undergo oxidation reactions, intramolecular cyclization and rearrangement to give various geranylhydroquinone and geranylbenzoquinone derivatives occurring from *Cordia* species woods [76].

**Fig. 1:**
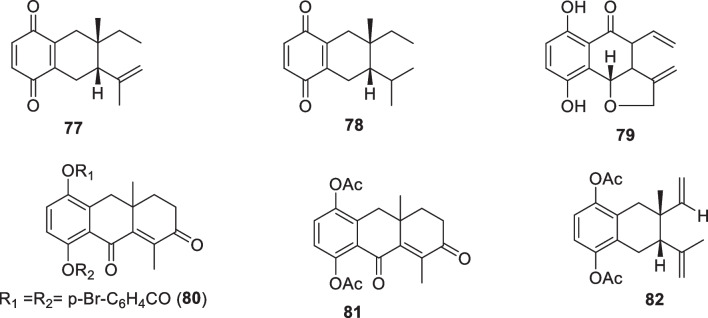
Synthesized quinone derivatives from the *Cordia* genus

Many syntheses have been done to elucidate the structures, suggest biosynthetic pathways of isolated quinones from Cordia species, and compare the biological activities of the different compounds. This latter had resulted in other quinone derivatives with biological activities. For instance, the structure of cordiachrome C (**3**) was confirmed by its hydrogenation in ethyl acetate after reoxidation to obtain dihydrocordiachrome C (**77**). After reoxidation, its hydrogenation in acetic acid afforded tetrahydrocordiachrome C (**78**) [72]. After the isolation of cordiachromes A–G (**1–6**, **60**), cordiachrome H (**79**) was obtained through oxidation of leucocordiachrome H (**61**) by silver oxide [75]. The absolute configuration of cordiaquinol I (**39**) was determined by adding (14 mg, 0.05 mmol) pyridine (4 mL) and *p*-bromobenzoylchloride (58 mg, 0.26 mmol) and stirring for 24 h at room temperature to afford 1,4-*p*-dibromobenzoylcordiaquinol I (**80**) [79]. Diacetylcordiaquinol I (**81**) was obtained through the addition of (8 mg, 0.03 nmol), pyridine (0.5 mL), and acetic anhydride (0.5 mL) to cordiaquinol I (**39**) [79]. Cordiaquinol C (**36**) (83 mg, 0.34 mmol), in the presence of pyridine (2 mL) and acetic anhydride (2 mL) afforded diacetylcordiaquinol C (**82**) [79] (Fig. 1).

The abundance of isolated quinones from *Cordia* species provides a wide range of pharmacological activities that can lead to new drug discovery.

## Biological studies and therapeutic potential

Prompted by ethnomedicinal uses of *Cordia* species in preventing and treating various diseases in traditional medicine [7, 8], various studies have been undertaken to shed light on the biological activity of extracts and isolated compounds.

### Cytotoxicity

Evaluation of the cytotoxic activities of cordiachromes [B (**2**), C (**3**)], cordiaquinol C (**36**), globiferin (**45**), alliodorin (**46**), and elaeagin (**66**), isolated from *C. globifera*, against KB (human epidermoid carcinoma of the mouth), BC-1 (human breast cancer cells), NCI-H187 (human small cell lung cancer), and Vero cell lines (African green monkey kidney fibroblast cells), were carried out. Compounds **2**, **3** and **36** exhibited activity against the cell lines mentioned above with IC_50_ values ranging from 0.2 μM to 6.9 μM, while globiferin (**45**) was active only against NCI-H187 cells with an IC_50_ value of 0.5 ± 0.04 μM [17].

The cytotoxicity of compounds **48** and **49** from *C. globosa* was evaluated in vitro against human colon adenocarcinoma (HCT-116), ovarian carcinoma (OVCAR-8) and glioblastoma (SF-295) cell lines. None showed antiproliferative effects at maximum concentrations of 20 μM [5].

Cordiaquinones B (**21**), E (**24**), L (**30**), N (**32**), and O (**33**) from *C. polycephala* roots were tested against HCT-8 (colon), HL-60 (leukemia), MDA-MB-435 (melanoma), and SF295 (brain) cancer cell lines [4]. All the compounds were active against all these cancer cell lines with IC_50_ values ranging from 1.2 to 11.1 μM, but compounds **32** and **33** were most active with IC_50_ values from 1.2 to 3.4 μM. Compound **21** was most active against HL-60 cells with an IC_50_ value of 2.2 μM (positive reference Doxorubicin with IC_50_ value = 0.02–0.8 μM) [4]. The authors suggested that the elevated activity of compounds **32** and **33** may be related to the presence of the α, β-conjugated carbonyl at the end of the tigloyloxy chain [4]. Chemical investigation of *C. globifera* led to the isolation of globiferane (**47**), which showed weak cytotoxicity against the following cell lines: HepG2 (human hepatocellular liver carcinoma), MOLT-3 (acute lymphoblastic leukemia), A549 (human lung carcinoma), and HuCCA-1 (human lung cholangiocarcinoma) with IC_50_ values of 148.6, 3.7, 148.6, and 66.0 μM, respectively, using an MTT (3-(4,5-dimethyl-2-thiazolyl)-2,5-diphenyl-2H-tetrazoliumbromide) assay [80]. Its derivative (1aS*,1bS*,7aS*,8aS*)-4,5-dimethoxy-1a,7a-dimethyl-1,1a,1b,2,7,7a,8,8a-octahydrocyclopropa[3,4]cyclopenta[1,2,b]naphtalene-3,6-dione (**50**) isolated from *C. globosa* roots exhibited significant cytotoxicity activity against colon (HCT-8), leukemia (HL-60, CEM), skin (B-16), and MCF-7 (breast) cancer cell lines, with IC_50_ values ranging between 1.2 and 5.0 μM [31]. The observed cytotoxicity exhibited by compound **(50)** may be due to the electron-donating methoxy groups on the aromatic ring. They are considered essential for anticancer activity [97]. According to Liew et al., compounds with a methoxy group substituted at C-2 of a quinone ring inhibit the growth of cancer cells. In addition, two or more methoxy substituents attached to its side showed more significant cytotoxicity [98].

Pessoa et al. evaluated the cytotoxicity of oncocalyxones A (**18**) and C (**59**) isolated from *C*. *oncocalyx* on human cell lines CEM (leukaemia), SW 1573 (lung tumour) and CCD922 (normal skin fibroblasts). Oncocalyxone A revealed toxicity with IC_50_ values of 0.76 ± 0.05, 7.0 ± 1.7 and 13.4 ± 0.6 μg/mL on CEM, SW 1573, and CCD922, respectively. Oncocalyxone B (**58**) also showed cytotoxicity with IC_50_ values of 1.5 ± 0.3, 7.5 ± 0.7 and 12.4 ± 0.5 μg/mL on CEM, SW 1573, and CCD922, respectively [93]. In addition, the cytotoxicity of oncocalyxone A (**18**) was evaluated against human normal [PBMC (peripheral blood mononuclear cells)] and tumoral [HL-60 (promyelocytic leukemia), SF-295 (glioblastoma), OVCAR-8 (ovarian carcinoma), and HCT-116 (colon carcinoma)] cell lines. It showed high cytotoxic activity on human leukemic cancer cells and normal leukocytes with IC_50_ values of 11.2 and 6.8 μM, respectively while exhibiting IC_50_ values above 16.5 μM against the remaining cell lines [85].

Moreover, Marinho-Filho et al. examined the cytotoxic effect of ( +)-cordiaquinone J (**28**) isolated from C. *leucocephala* on tumor cells. In an MTT assay, ( +)-cordiaquinone J (**28**) demonstrated cytotoxicity activity after 72 h of incubation against HL-60 (leukemia), HCT-8 (colon), SF295 (brain), MDA-MB-435 (melanoma), and normal PBMC (Lymphocytes) with IC_50_ values of 2.7 μM, 4.9 μM, 6.6 μM, 5.1 μM, and 10.4 μM, respectively compared to doxorubicin as a positive control with IC_50_ 0.03 μM, 0.02 μM, 0.4 μM, 0.8 μM, and 1.7 μM, respectively [90].

The cytotoxicity of compounds **1, 2, 3, 36, 39, 40, 41,** and **46** isolated from *C. fragrantissima* and their synthesized analogues (**80**, **81,** and **82**) against COS-7 (African green monkey kidney cells, epithelial-like) and HUH-7 (Human liver cancer cells, epithelial-like) were inactive in an XTT assay compared to MG 132 (carbobenzoxy-l-leucyl-l-leucyl-l-leucinal) used as reference [79].

Previous biological studies reported that the cytotoxic activity of quinones is due to their ability to react as dehydrogenating and oxidizing agents [20]. The cytotoxicity of quinones can also be explained by their capacity to inhibit electron transporters [99], protein adduct formation [100], oxidative phosphorylation [101], and reactive oxygen species (ROS) production [102] as well as through enzyme SH groups and direct DNA damage [39, 90].

### Antifungal and larvicidal activities

Ioset et al. evaluated the antifungal and larvicidal activities of cordiaquinones B (**21**), E (**24**), F (**25**), G (**26**), and H (**27**) isolated from *C. linnaei* using TLC bioautographic and agar–dilution assays [81]. The compounds (**21**, **24–26)** were active against *Candida albicans* and *Dosporium cucumerinum* with minimum inhibitory concentrations (MIC) ranging from 0.5 to 6 μM compared to nystatin (0.2–1.0 μM) used as a positive reference. However, compound **27** was inactive on both fungi. Its inability to inhibit the bacterial strains might be due to an epoxide [81]. Regarding their larvicidal potential, all the compounds showed activity against *Aedes aegypti* with MIC values between 12.5 and 50 μg/mL compared to reference plumbagin (MIC = 6.25 μg/mL), except for compound **27,** which was not tested [81].

2-(2Z)-(3-Hydroxy-3,7-dimethylocta-2,6-dienyl)-1,4-benzenediol (**52**), isolated from the roots and bark of *C. alliodora*, exhibited weak activity against *Cladosporium cucumerinum* in bioautography and in agar-dilution assays with an MA (Minimum amount to inhibit growth on the SiO_2_ gel TLC) value of 5 μg and MIC of 15 μM respectively. This compound was inactive against *C. albicans* on TLC bioautography, and consequently, it was not tested by agar–dilution assay [27].

Cordiaquinones A (**20**), J (**28**), and K (**29**) showed antifungal activity against *C. cucumerinum* and *C. albicans* in bioautographic and agar-dilution assays with similar values (MA = 0.5 μg and MIC = 3 μg/mL) as the reference drug nystatin (MA = 0.1 μg and MIC = 1 μg/mL). These compounds also demonstrated weak larvicidal effects on *Aedes aegypti* with MIC values of 12.5—25 μg/mL [28].

The antifungal activity of ehretiquinone (**35**), isolated from *C. anisophylla*, was evaluated on *C. albicans* (DSY262 and CAF2-1 strains) using bioautography, agar–dilution assays and mature biofilm [91]. The compound was more active against strain DSY262 with a minimum inhibition quantity (MIQ) ≤ 5 μg compared to CAF2-1 with a MIQ of 25 μg. However, the compound (**25)** was inactive in the agar–dilution assay and mature biofilm [91].

Dettrakul et al. investigated the antifungal activity of cordiachrome B (**2**) and C (**3**), isolated from *C. globifera*. Both compounds exhibited weak antifungal activity against *C. albicans* with IC_50_ values of 7.7 μM and 4.6 μM, respectively, whereas globiferin (**45**), cordiaquinol C (**38**), and alliodorin (**46**) were inactive with IC_50_ values > 20 μM (positive control amphotericin B, IC_50_ = 0.08 μM) [17]. The antifungal activity of oncocalyxone A (**18**) done by Silva et al. showed that it did not inhibit the growth of tested fungi (*C. albicans* ATCC 10234^™^, *C. neoformans* ATCC 48184™, *A. fumigatus* ATCC 13073^™^, *S. schenckii* ATCC 201679^™^ and *T. interdigitale* 73896) with MIC values > 151 μg/mL [103].

### Antileishmanial activity

The chemical investigation of *C. fragrantissima* wood extract led to the isolation of several cordiaquinols (**36, 39, 40,** and **41**), cordiachromes (**1, 2,** and **3**) and alliodorin (**46)** [73, 79]. The authors also synthesized related compounds, 1,4-*p*-dibromobenzoylcordiaquinol I (**80**), acetylcordiaquinol I (**81**), and acetylcordiaquinol C (**82**) [79]. All the compounds, including their derivatives, were assayed for antileishmanial assay against promastigote forms of *Leishmania major*, *L. panamensis,* and *L. guyanensis* using an MTT assay [79]. All the compounds were active with IC_50_ values of 1.4–81.4 μM were found more active on *L. panamensis* and *L. guyanensis* than *L. major,* while compounds **1**, **2**, **36**, **40**, **46,** and **82** exhibited good activity against *L. major* with IC_50_ values of 4.1, 2.5, 4.5, 2.7, 7.0, and 1.4 μM, respectively, compared to Amphotericin B (IC_50_ less than 0.1 μM) used as a positive control [73, 79].

In related studies, cordiaquinone E (**24**), isolated from the roots of *C. polycephala*, was evaluated for its activity against promastigote and axenic-amastigote forms of *L. amazonensis *in vitro*.* The compound inhibited the growth of the promastigote form with an IC_50_ value of 4.5 ± 0.3 μM as well as against the axenic-amastigote form with 2.89 ± 0.11 μM, with selectivity indexes (SI) of 54.84 and 85.4, respectively. The evaluation of cordiaquinone E (**24**) against intracellular amastigotes was carried out to support the notion of antileishmanial activity. It led to a better result with an EC_50_ value of 1.92 ± 0.2 μM and an SI of 128.54 using an MTT assay. The growth inhibition assay of compound **24** on RAW 264.7 macrophages led to a CC_50_ value of 1246.81 ± 14.5 μM. Antileishmanial activity of compound **24** on *L. amazonensis* was evaluated using Amphotericin B [IC_50_ 0.35 ± 0.05 μM (promastigote form); IC_50_ 0.51 ± 0.02 μM (axenic-amastigote form)] and Meglumine antimoniate [IC_50_ 21,502 ± 481 μM (promastigote form); IC_50_ 1730 ± 33.5 μM (axenic-amastigote form)], as reference drugs respectively [89]. Rodrigues et al. explained the antileishmanial activity of cordiaquinone E. Firstly, by apoptosis, which associates externalization of phosphatidylserine and necrotic cell death, and secondly, by immunomodulation [89].

### Anti-inflammatory activity

Five meroterpenoids (**15, 38, 42, 43,** and **44**) isolated from *C. glazioviana* were evaluated for their anti-inflammatory activity against RAW 264.7 macrophage murine cells through cellular viability and lipopolysaccharide (LPS) induction. The cytotoxicity of isolated compounds was evaluated by MTT assay [34]. Rel-1,4-dihydroxy-8α,11α,9α,11α-diepoxy-2-methoxy-8aβ-methyl-5,6,7,8,8a,9,10,10a-octahydro-10-antracenone (**15**), cordiaquinol E (**38**), 10,11-dihydrofuran-1,4-dihydroxyglobiferin (**42**), 2-[(1ʹE,6ʹE)-3ʹ,8ʹ-dihydroxy-3ʹ,7ʹ-dimethylocta-1ʹ,6ʹ-dienyl]-benzene-1,4-diol (**43**), and 6-[(2ʹR)-2ʹ-hydroxy-3ʹ,6ʹ-dihydro-2H-pyran-5ʹ-yl]-2-methoxy-7-methylnaphthalene-1,4-dione (**44**) induced inflammation against RAW 264.7 macrophage cells by reducing cells viability with IC_50_ range value 71.66 ± 15.44–609.48 ± 5.05 μM. Lipopolysaccharide production was evaluated by inducing oxide nitric in RAW 264.7 cells. Among these compounds, 10,11-dihydrofuran-1,4-dihydroxyglobiferin (**42**) exhibited the best inhibition of NO (Nitric Oxide) synthesis with IC_50_ 50.34 ± 9.88 μM, followed by compounds **44** (66.73 ± 10.28 μM) and **43** (105.83 ± 5.09 μM); the rest produced weak inhibition to induced inflammation against RAW 264.7 macrophage compared to dexamethasone (IC_50_ 1.79 ± 0.04 μM) used as a positive control [34].

Ferreira et al. examined the anti-inflammatory activity of the water-soluble fraction of the heartwood methanolic extract of *C. oncocallyx*. The quinone fraction containing mainly oncocalyxone A (**18**) was very active in inhibiting paw edema induced by a carrageenan injection, with a 57% and 60% reduction three hours after a dose of 10 and 30 mg/kg body weight, respectively [104].

### Antimicrobial, antibiofilm, antimycobacterial and antioxidant activities

Previous biological evaluation of *C. oncocalyx* revealed that oncocalyxone A (**18**) could inhibit the growth of Gram-positive and Gram-negative pathogenic strains, even clinical specimens. It was more sensitive to *Staphylococcus* species than to *Enterococcus*, *Listeria*, *Acinetobacter,* and *Stenotrophomonas* species with an MCI range from 9.43 μg/mL to 151 μg/mL, and it showed high sensitivity against *S. epidermidis* (ATCC 12228™) with MIC 9.43 μM compared to vancomycin (MCI 1 μM) used as reference[103]. It also inhibited the growth of *S. aureus* MED 55 (MIC 18.87 μM), *S. aureus* COL and *S. epidermidis* 70D (MIC 37.75 μM); and *E. faecalis* ATCC512999^™^ (MIC 75.5 μM) [103]

It showed inhibition of biofilm production by ⁓70% in methicillin-resistant *S. aureus* MED 55 strain (resistant clinical specimen) [103]

Khan et al. examined the antimicrobial and antioxidant activities of the GC–MS profile fractions of *C. rothii* roots*.* The *n-*hexane fraction, which contained cordiachrome C (**3**), exhibited weak antibacterial activity against Gram-positive and Gram-negative bacteria. While the MeOH marc extract containing cordiaquinol C (**36**) and cordiachromene A (**57**) showed good antibacterial activity against *Staphylococcus epidermidis* with a minimum inhibitory concentration (MIC) 250 μg/disk, EtOAc marc extract containing cordiol A (**55**) was inactive against all the tested bacteria [42].

Regarding the antioxidant activity of these extracts, MeOH and EtOAc marc left extract of *C. rothii* roots have good activity with EC_50_ 93.75 μM than *n*-hexane extract, which showed weak activity with EC_50_ 187.5 μM [42].

Previous biological studies examined the antioxidant activity of the methanol extract of the heartwood of *C. oncocalyx*. The quinone fraction (80% oncocalyxone A (**18**)) was evaluated in a rat model with CCl_4_-induced hepatotoxicity and the prolongation of pentobarbital sleeping time in mice by measuring plasma GPT and GOT. Only the quinone fraction inhibited the GPT level significantly (29%) with a 30 mg/kg dose. It also caused a significant reduction (45%) of CCl_4_-induced prolongation of pentobarbital sleeping time with a dose of 10 mg/kg. It confirmed the hepatoprotective effect involving free radical and lipoperoxidation and correlated with the antioxidant properties of quinones [105]. The latter is possibly due to the presence of oncocalyxone A, the main constituent [106]. Moreover, quinones are renowned for redox cycling ability [107]; this is related to their free radical scavenging activity which promotes their antioxidant activity [108].

In addition, cordiachrome C (**3**) and globiferin (**45**) showed significant antimycobacterial activity with MIC 1.5 and 6.2 μg/mL, respectively, while cordiachrome B (**2**) (12.5 μg/mL), cordiaquinol C **(36**) (25.0 μg/mL), diacetylcordiaquinol C (**82**) (25.0 μg/mL), alliodorin (**46**) (12.5 μg/mL), and elaeagin (**66**) (12.5 μg/mL) displayed weak activity compared to Rifampicin (0.0047 μg/mL), Isoniazid (0.05 μg/mL), and Kanamycin (2.5 μg/mL) used as standard drugs [17].

### Antimalarial and hemolytic activities

Cordiachrome C (**3**), cordiaquinol C (**36**), and diacetylcordiaquinol C (**82**) were evaluated for antimalarial activity against *Plasmodium falciparum* using dihydroartemisinin (IC_50_ 0.0012 μg/mL), used as reference*.* They exhibited significant activity with IC_50_ 0.2 ± 0.1 μg/mL, 0.3 ± 0.0 μg/mL, and 0.4 ± 0.1 μg/mL respectively, more than cordiachrome B (**2**) (IC_50_ 1.5 ± 0.2 μg/mL), globiferin (**45**) (IC_50_ 2.1 ± 0.5 μg/mL), alliodorin (**46**) (IC_50_ 3.1 ± 0.5 μg/mL), and elaeagin (**66**) (3.6 ± 0.1 μg/mL) [17].

Silva et al. evaluated the hemolytic activity of oncacalyxone A (18) through erythrocyte damage due to hemoglobin release. The compound did not show activity at the tested concentrations ≥ 151 μg/mL [103].

Compounds **21**, **24**, **30**, **32,** and **33** from C. *polycephala* roots were evaluated for hemolytic activity in mice erythrocytes. None was active with EC_50_ > 500 μmol L^−1^ [4].

### Neuroinhibitory effect

Matos et al. (2017) examined the neuroinhibitory effect of different compounds (**9**–**18**) isolated from *C. oncocalyx* by mice vas deferens bioassay. Compounds **10, 11** and **14** significantly inhibited the neurogenic contraction by 76%, 69%, and 63%, respectively, whereas compounds **12** and **15** did not considerably affect neurogenic contraction. Compounds **9**, **10**, **14**, **16, 17** and **18** showed a completely reversible neuroinhibitory effect upon adding the pharmacological antagonist Promethazine and a partial reversible effect by yohimbine. Neurogenic contraction induced by compound **11** was irreversible by adding naloxone, famotidine, promethazine or yohimbine antagonists. However, compounds **9**, **10**, **14**, **16**, **17** and **18** did not inhibit neurogenic contractions using the ODQ, famotidine or naloxone antagonists. The authors found that reversible action may be related to pre-synaptic terminal and pre-synaptic receptor inhibition due to the co-release of histamine and norepinephrine [32].

Although previous reviews reported different isolation methods and biological activities of *Cordia* quinones, we noted a lack of information that could help to valorize them. We suggest that future research should focus on the structure–activity relationships and mechanisms of action of the quinones of the genus *Cordia.* More in vivo biological tests and clinical studies should be performed. Up to now, just one clinical study has been done on *Cordia* quinones (cordiachrome F for allergenic). To improve the number of quinones isolated from *Cordia* species, pressurized liquid extraction (PLE) could be used. [109]. Pressurized hot water extraction to optimize the extraction of volatile components [110] and dry extraction to enrich powder fractions with an extensive range of secondary metabolites could also be done. [111, 112].

## Conclusion

Using Cordia species in traditional medicine to treat various diseases has increased interest in their phytochemistry. This review presents the collective phytopharmacological information on *Cordia* quinones from 1972 to 2023. The research shows that over 70 (**1–70**) quinones have been isolated from different parts of *Cordia* species with different skeletal structures. Meroterpenoid quinones were the major class of compounds isolated, with meroterpenoid benzoquinones being the most predominant in most species. The biosynthesis of *Cordia* quinones is not yet well understood, but the biogenesis and some biosynthetic pathways have been proposed to explain the presence of quinones in the *Cordia* genus.

The extracts and isolated quinones demonstrated antimalarial, antimicrobial, anti-inflammatory, antibiofilm, antioxidant, antimycobacterial, antileishmanial, larvicidal, hemolytic, neuroinhibitory, and cytotoxicity properties. Most studies reported cytotoxicity against particularly cancer cell lines. It may be due to the ethnomedicinal uses of these species and the anticancer properties of the quinones. Although the biological activities of compounds can often be related to their structures, there is currently little information available to explain structure–activity relationships for the quinones occurring in *Cordia* species. This review discussed the potential of the genus *Cordia* as a promising source of new bioactive compounds that can provide quinones for various pharmaceutical applications.

## Data Availability

All data are included in the manuscript.

## Abbreviations

AC_2_O: Acetic anhydride
CCl_4_: Tetrachloromethane
CHCl_3_: Chloroform
CH_2_Cl_2_: Dichloromethane
DMSO: Dimethyl sulfoxide
EtOH: Ethanol
GOT: Glutamate-oxalate-transaminase
GPT: Glutamate-pyruvate-transaminase
ODQ: Soluble guanylate cyclase inhibitor
RP-HPLC: Reverse phase high-performance liquid chromatography
MeOH: Methanol
Na_2_S_2_O_4_: Sodium dithionite
MTT: 3-[4,5-Dimethylthiazole-2-yl]-2,5-diphenyl-tetrazolium bromide
THF: Tetrahydrofuran
XTT: 2,3-Bis-(2-methoxy-4-nitro-5-sulfophenyl)-2H-tetrazolium-5-carboxanilide
v/v: Volume by volume
1D and 2D: One dimension and two dimensions
